# Simulated Winter Incubation of Soil With Swine Manure Differentially Affects Multiple Antimicrobial Resistance Elements

**DOI:** 10.3389/fmicb.2020.611912

**Published:** 2020-12-16

**Authors:** Daniel N. Miller, Madison E. Jurgens, Lisa M. Durso, Amy M. Schmidt

**Affiliations:** ^1^USDA-ARS, Agroecosystem Management Research Unit, Lincoln, NE, United States; ^2^University of Nebraska, Lincoln, NE, United States; ^3^Department of Biological Systems Engineering, University of Nebraska, Lincoln, NE, United States

**Keywords:** antibiotic resistance, freeze-thaw, manure application, manure treatment, soil moisture, swine

## Abstract

Gastrointestinal bacteria that harbor antibiotic resistance genes (ARG) become enriched with antibiotic use. Livestock manure application to cropland for soil fertility presents a concern that ARG and bacteria may proliferate and be transported in the environment. In the United States, manure applications typically occur during autumn with slow mineralization until spring planting season. A laboratory soil incubation study was conducted mimicking autumn swine manure application to soils with concentrations of selected ARG monitored during simulated 120-day winter incubation with multiple freeze-thaw events. Additionally, the effects of two soil moistures [10 and 30% water holding capacity (WHC)] and two manure treatments [raw versus hydrated lime alkaline stabilization (HLAS)] were assessed. Fourteen tetracycline resistance genes were evaluated; *tet*(D), *tet*(G), and *tet*(L) were detected in background soil while swine manure contained *tet*(A), *tet*(B), *tet*(C), *tet*(G), *tet*(M), *tet*(O), *tet*(Q), and *tet*(X). By day 120, the manure-borne *tet*(M) and *tet*(O) were still detected while *tet*(C), *tet*(D), *tet*(L), and *tet*(X) genes were detected less frequently. Other *tet* resistance genes were detected rarely, if at all. The sum of unique *tet* resistance genes among all treatments decreased during the incubation from an average of 8.9 to 3.8 unique *tet* resistance genes. Four resistance elements, *int*I1, *bla*_*ctx–m–*32_, *sul*(I), *erm*(B), and 16s rRNA genes were measured using quantitative PCR. ARG abundances relative to 16S abundance were initially greater in the raw manure compared to background soil (−1.53 to −3.92 log abundance in manure; −4.02 to <−6.7 log abundance in soil). In the mixed manure/soil, relative abundance of the four resistance elements decreased (0.87 to 1.94 log abundance) during the incubation largely because 16S rRNA genes increased by 1.21 log abundance. Throughout the incubation, the abundance of *int*I1, *bla*_*ctx–m–*32_, *sul*(I), and *erm*(B) per gram in soil amended with HLAS-treated manure was lower than in soil amended with raw manure. Under low initial soil moisture conditions, HLAS treatment reduced the abundance of *int*I1 and resulted in loss of *bla*_*ctx–m–*32_, *sul*(I), and *erm*(B)] compared to other treatment-moisture combinations. Although one might expect antibiotic resistance to be relatively unchanged after simulated winter manure application to soil, a variety of changes in diversity and relative abundance can be expected.

## Introduction

Manure from animal production is a valuable source of fertilizer nutrients and organic matter for crop production offsetting the need for chemical fertilizer inputs and enhancing many soil attributes (Edmeades, 2003). However, manure management presents potential challenges, including odors, runoff, greenhouse gas emissions, and pathogens when best management practices are not used. Impairment of surface waters with zoonotic pathogens that may be associated with manure application have lent more focus toward pathogen control/inactivation prior to and during manure application to cropland. Contributions from livestock manure to antibiotic resistance in environmental matrices presents a growing concern about the fate of resistant microorganisms or the dissemination of antibiotic resistance genes (ARG) during manure treatment or following manure application to soil (Durso and Cook, 2014; Topp et al., 2018). The use of manure as a soil and crop fertility input could represent an important contribution of antibiotic resistant bacteria (ARB) and ARG to the soil environment (Franklin et al., 2016).

Croplands receiving manure application contain indigenous soil microorganisms that normally harbor a low, yet diverse, abundance of background of ARG (Durso et al., 2016). Once in the soil environment, ARGs originating from animal manures or human biosolids could potentially move into indigenous soil microbial populations though horizontal gene transfer of mobile genetic elements (von Wintersdorff et al., 2016), which are often found on particularly stable plasmids that increase the chance of inheritance between species (Heuer et al., 2009). Once acquired, the low fitness cost of antibiotic resistance enables soil bacteria harboring ARGs to persist in the environment even though antibiotic pressures are no longer present (Cook et al., 2014). Although this chain of events seems exceedingly rare, Forsberg et al. (2012) identified non-pathogenic soil microorganisms containing several long resistance genes having 100% nucleotide identity to those in human pathogens indicating a possible recent transfer event.

The abundance of specific ARGs may increase, decrease, or remain unchanged overtime with continuous/long term manure inputs (Chee-Sanford et al., 2009; Mantz et al., 2013; Peng et al., 2015; Tang et al., 2015; Durso et al., 2018; McKinney et al., 2018), indicating the fate of ARB and ARG in soils is likely dependent upon repeated manure inputs and a multitude environmental factors. Proliferation of indigenous ARB with the influx of manure nutrients and substrates can enhance ARB persistence (Heuer et al., 2011; Jechalke et al., 2013; Udikovic-Kolic et al., 2014; Fang et al., 2015) but may not be as important as the manure microbes themselves (Peng et al., 2016). Manure source (cattle, swine, or poultry), environmental factors (soil type, moisture and temperature), and management decisions (tillage practices, application timing, and method, and location of manure application) also likely influence persistence of ARG in soils.

In confined swine production systems, manure is typically collected and stored (with or without treatment) for a period of 6 months to a year to reduce the occurrence of pathogens (Hill, 2003; McLaughlin et al., 2012). During manure storage/treatment, biological, chemical, and physical processes reduce pathogen load and provide some capacity to reduce, but not eliminate, ARGs (Joy et al., 2014). Untreated human biosolids have many of the same characteristics as swine manure, and a preferred method to reduce pathogen loads in human biosolids is through alkaline stabilization using hydrated lime to raise the pH of the biosolids for a sustained period of time (US Environmental Protection Agency, 2000).

Recently, a hydrated lime alkaline stabilization (HLAS) manure treatment, modeled after the alkaline stabilization used on human biosolids, has been shown to reduce porcine epidemic diarrhea virus (PEDV), an important coronavirus disease in swine (Stevens et al., 2018). Research investigating the impact of HLAS manure treatment on ARG abundance is limited; however, a survey of three resistance genes [*tet(*W*), tet(*O*)*, and *sul(*I*)*] in manure, soil, human biosolids, and lime-stabilized human biosolids found lower abundances of these ARG in lime-stabilized biosolids (Munir and Xagoraraki, 2011). The long-term impact of manure or biosolids application on soil microbial communities in this study was less clear, with considerable farm-to-farm variability. It should be noted that soil pH is an important driver of microbial community structure (Lauber et al., 2009; Tan et al., 2020). Indeed, agricultural lime addition to soils, a practice used to raise the pH of acidic soils, also affects soil community structure (Barth et al., 2018; Lin et al., 2018) and may impact diversity and abundance of soil ARG.

In swine production areas across the midwestern United States, stored manure slurry is land applied in the fall because manure nutrients are more easily retained in the cold soil over winter and available for new crop growth in the spring. Winter application of manure is discouraged due to contaminant runoff concerns resulting from frozen, snow-covered or saturated soil. Two manure applications methods are commonly used depending upon the solids content of the manure: surface and sub-surface application. Surface application via irrigation is common where manure is stored in treatment lagoons, as the very low solids content in lagoon effluent does not interfere with spray nozzle performance. An alternative storage method used in many swine production systems to minimize manure volume and retain valuable nitrogen for crop fertilization is collection in deep pits below the production area. This “slurry” manure has higher solids content and greater concentration of nutrients and is typically injected in narrow bands into crop soils to minimize nitrogen losses via volatilization of ammonia. Manure injection produces a unique microenvironment where manure microbes and nutrients are in close contact with soil microbes. During application of liquid or slurry manure application, soil moisture also increases based upon manure water content, soil characteristics and hydrologic conditions. Application of manure to saturated soils is both difficult and discouraged as it contributes to manure runoff. Following fall manure application, low temperatures and soil freeze-thaw during winter months may impact bacterial competition, survival, and persistence of organisms and ARG.

This study examines how ARGs indigenous to either agricultural soil or swine manure change after simulated manure application and 120-day winter soil incubation with multiple freeze-thaw events, with and without manure HLAS treatment. We hypothesized that (1) HLAS treatment would have a strong immediate effect on resistance by directly limiting the survival of microorganisms; however, (2) few changes in diversity or persistence of ARG would occur over time under “winter” incubation conditions. Additionally, the effects of two initial soil moisture contents were assessed. Fourteen different tetracycline resistance genes were evaluated, and the abundance of four resistance elements both in bulk soil and relative to 16s rRNA gene abundance were measured using qPCR molecular techniques.

## Materials and Methods

### Soil and Manure Treatments and Incubations

A silty clay loam soil was collected from a crop field in eastern Nebraska, sieved, and air dried (soil properties: 5.45 μg g^–1^ NO_3_-N, 2.43 μg g^–1^ NH_4_-N, 0.23 dS m^–1^ EC, 7.00 pH, 63.03 μg P g^–1^ Mehlich P). The WHC of the soil was determined gravimetrically (Klute, 1986) and two stock soil mixtures were prepared by thoroughly incorporating deionized water to be achieve 10 or 30% of the maximum WHC of the soil (i.e., “dry” and “moist” soil conditions, respectively). Expressed on a moisture content basis, the 10 and 30% WHC soils contained 82.6 and 247.7 g H_2_O kg^–1^ dry soil, respectively. From each soil mixture, 30 g (oven dry equivalent) soil was added to multiple 50 mL tubes. A soil cavity (simulated manure furrow) was made in the center of each soil tube by pressing a 10 mL pipet tip into the soil and tubes were immediately capped to prevent moisture loss.

**FIGURE 1:**
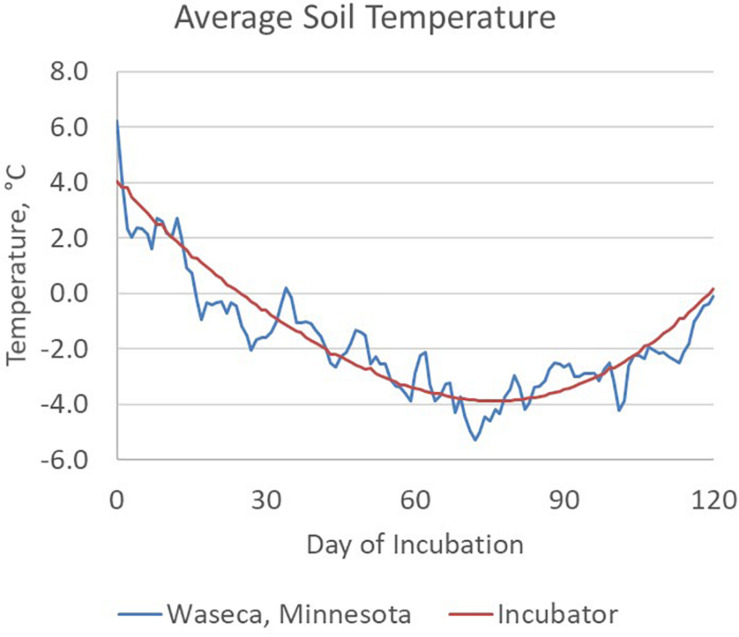
Average soil temperature between November 11th and March 11th calculated for a 6-year period (2009 – 2014) of field data at the Waseca, MN experimental station. Two freeze/thaw cycles were conducted each week during the first 5 weeks of the incubation, typical for surface soils at this location.

Manure slurry was collected from the deep pit of a commercial swine production site in south central Nebraska and refrigerated at 4°C for less than 48 h prior to setting up the soil incubation. The day prior to setting up the incubations, manure slurry was divided into two stocks and equilibrated to room temperature (20°C). One manure slurry stock was amended with hydrated quicklime (10 g L^–1^ of manure slurry) yielding a final, stable pH of 11.5 and designated HLAS-treated. Although a higher pH of 12 for 2 h is recommended for biosolids treatment (US Code of Federal Regulations, 2018), an earlier study demonstrated that pH 10 for 12 h effectively controlled PEDV (Stevens et al., 2018). The other manure slurry stock (Raw) received no quicklime treatment. Both treatments were incubated at room temperature overnight since effective HLAS treatment recommends at least a 6-h exposure time at high pH. On day 0, 80 soil incubations were prepared by adding 10 g of manure slurry to the soil cavity in each vial yielding 20 replicates of each moisture (10 and 30% WHC) and manure (HLAS and raw manure) combination. Four samples of each treatment combination were immediately stored at −80°C as Day 0 samples. All other samples were capped loosely, secured in tube racks, and randomly placed in a refrigerated incubator (Fisherbrand Isotemp BOD Refrigerated Incubator, Fisher Scientific, Waltham, MA, United States). The incubator temperature was adjusted daily to simulate mean winter soil temperatures (5.1 cm depth) at the University of Minnesota Southern Research and Outreach Center in Waseca, Minnesota (Figure 1). The Waseca location was selected based upon the extensive soil temperature (daily high and low) records, Waseca’s location in an area of dense swine production, and because seasonal temperature changes spanned the range where soils froze for extended periods of time. Twice weekly during the first 10 weeks of incubation, the racks of tubes were removed from the incubator and either allowed to briefly (over 20 min) freeze in the −80°C freezer (if the current incubation temperature was above 0°C) or thaw at room temperature (if the current incubation temperature was below 0°C). The number of freeze/thaw events was selected based upon the number of freeze/thaw events observed within November, December, and March at 5 cm soil depth in soils at Waseca, MN in 2009, 2011, 2012, 2013, and 2014, and ranged from six in 2013 to 22 in 2011. At Days 30, 60, 90, and 120, four samples of each treatment combination were retrieved and stored at −80°C for subsequent DNA extraction.

### DNA Extraction, Multiplex PCR, and Quantitative PCR Amplification

Immediately prior to DNA extraction, deionized water was thoroughly mixed into each manure/soil sample to yield a uniform sample moisture content (25% moisture content wet basis) since incubation tubes lost moisture over the 120-day incubation (determined gravimetrically). DNA was extracted from 250 mg of slurry using the PowerSoil DNA Isolation Kit (MoBio Laboratories, Solana Beach, CA, United States) according to the manufacturer’s protocol. DNA extracts were stored at −80°C until polymerase chain reaction (PCR) analysis.

A variety of target genes were assessed to gage how manure treatments and simulated winter incubation conditions affected resistance (Table 1). These target genes included “highly” and “critically important” resistance genes conferring resistance to antibiotics used in animal production. Four multiplex PCR amplification reactions for the presence/absence of fourteen tetracycline resistance genes (Table 2) were performed on each DNA extract according to the method of Ng et al. (2001), with each 25-μL reaction containing 12.5 μL Jumpstart RedTaq Master Mix and 0.5 μL of each primer. Extractions were processed in a StepOnePlus Real-Time PCR System (Applied Biosystems, Waltham, MA, United States) at settings of 5 min at 94°C; 35 cycles of 1 min at 94°C, 1 min at 55°C, 90 s at 72°C; and 5 min at 72°C. Positive controls and blanks were included in each PCR run, and PCR product was separated using agarose gel, stained (ethidium bromide), and photographed (UVP GelDoc-It TS3 Imager, Upland, CA, United States) to visualize PCR amplification products and confirm PCR product sizes.

**TABLE 1:** List of target genes.

Gene	Function*
*int*l1	Encodes for an integron-integrase gene that helps antibiotic resistance genes spread from cell to cell. It has been proposed as a gene that will help identify resistance that is associated with human activities (Gillings et al., 2015), and as a marker for “pollutants” including antibiotic resistance, heavy metals, and disinfectants.
*sul(*I*)*	Encodes for sulfonamide-resistance. One of the most studied resistance genes in environmental samples. Sulfonamides are classified as “Highly Important” (the second category) by the World Health Organization (WHO, 2018). Three drugs in this class are used in food animals and administered to groups of animals via food and water.
*bla*_*CTX–M*–32_	Encodes for third-generation cephalosporin resistance, one type of β-lactamase resistant drug. These drugs are classified as “Critically Important” (the top category) by World Health Organization. Most individual drugs in the class are limited to use on humans, pets (dog/cat), and horses, however, two (cefquinome and ceftiofur) are indicated for use in food animals, though they are not administered to groups of animals via food or water.
*erm*(B)	Encodes for resistance to macrolide drugs, such as erythromycin. These drugs are classified as “Critically Important” (the top category) by the World Health Organization. Erythromycin is used in large and small animals, is FDA approved for use in cattle, swine, and poultry, and is administered to food animals via food and water. A related macrolide, azithromycin, is also approved for use in sick food animals, and is individually listed on the CDC Antibiotic Resistance Threat list as a concern for some foodborne pathogens (CDC, 2019).
*tet*	Encodes for resistance to tetracycline drugs, including tetracycline, chlortetracycline, oxytetracycline, and doxycycline. The tetracyclines are the most widely used drugs in food animal production both globally, and in the United States. The tetracyclines account for 36% of all drugs sold in the United States in 2018 for veterinary purposes, with 44% of those attributed to use in cattle and 48% in swine (US Food and Drug Administration, 2019). Although these drugs are classified as “Highly Important” (the second category) by the World Health Organization, they are not listed on the CDC Antibiotic Resistance Threats in the United States 2019 publication.

**TABLE 2:** Quantitative PCR primers and thermocycler conditions utilized in this study.

PCR Target	Primer name	Primer sequence	Thermocycler program	Source
16s rRNA	TB 331F-16srRNA	5′-TCCTACGGGAGGCAGCAGT-3′	95°C (15 min)	Denman and McSweeney (2006)
	TB 518R-16srRNA	5′-ATTACCGCGGCTGCTGG-3′	35 cycles of 95°C (15 s), 55°C (20 s), 72°C (10 s)	
*erm(B)*	ermB F TB	5′-GGTTGCTCTTGCACACTCAAG-3′	94°C (4 min) 5 cycles* of 94°C (30 s), 63–58°C (30 s), 72°C (60 s)	Koike et al. (2010)
	ermB R TB	5′-CAGTTGACGATATTCTCGATTG-3′	30 cycles of 94°C (30 s), 58°C (30 s), 72°C (45 s) 72°C (7 min)	
*intI1*	TB intI1LC5 FW	5′-GATCGGTCGAATGCGTGT-3′	95°C (15 min)	Barraud et al. (2010)
	TB intI1LC1 RV	5′-GCCTTGATGTTACCCGAGAG-3′	40 cycles of 95°C (15 s), 55°C (30 s), 72°C (10 s)	
*sul(I)*	SμLI FW	5′-CGCACCGGAAACATCGCTGCAC-3′	95°C (15 min)	Pei et al. (2006)
	SμLI RVS	5′-TGAAGTTCCGCCGCAAGGCTCG-3′	50 cycles of 95°C (15 s), 65°C (30 s), 72°C (30 s)	
*bla*_*ctx–m–32*_	TB ctx-m-32FWD	5′-CGTCACGCTGTTGTTAGGAA-3′	95°C (15 min)	Szczepanowski et al. (2009)
	TB ctx-m-32RVS	5′-CGCTCATCAGCACGATAAAG-3′	35 cycles of 95°C (15 s), 63°C (30 s), 72°C (10 s)	

*Touchdown cycle decrease annealing temperature by 1°C/cycle. Standard curve *R*^2^ ranged from 0.960 to 0.998. Efficiency ranged from 80 to 117%.

Quantitative PCR reaction amplifications of 16s rRNA, *intI1, sul(*I*), erm(*B*)*, and *bla_*ctx–m–*32_* were performed according to Table 2. Standards were prepared from serial dilutions of 4-gene multiplex with 16s rRNA gene (Integrated DNA Technologies, Coralville, IA, United States). Each 5-μL volume of standard contained from 10^1^ to 10^8^ gene copies. Standards and reagent blanks were included in each run. All samples were quantified in triplicate and averaged. Standard curve R^2^ ranged from 0.960 to 0.998 while efficiency ranged from 80 to 117%.

### Statistics

Equality of proportions tests and ANOVA were performed using SAS statistical software (SAS Institute, Cary, NC, United States). The two-sample test of equality of proportions was chosen to generate probabilities related to the significance of initial soil moisture and manure treatment on the percent of each tetracycline resistance gene. Analysis of variance was used to evaluate the significance of antecedent soil moisture and manure treatment on (i) the diversity of tetracycline resistance genes present over time (ii) the log abundance of *intI1, sul(*I*), bla_*CTX–M–*32_*, and *erm(*B*)* in soil, and (iii) the log abundance of of *intI1, sul(*I*), bla_*CTX–M–*32_*, and *erm(*B*)* normalized to 16s rRNA gene abundance. A *P*-value of <0.05 was considered significant.

## Results

### Initial Resistances in Manure and Soil

Eight *tet* resistance genes were detected in the raw manure while background soil revealed three *tet* resistance genes (Table 3). There was no difference in the detection of *tet* resistance genes between HLAS-treated and raw manure. The *tet(*G*)* gene was detected in both soil and manure samples, while *tet(*E*), tet(*K*), tet(*S*)*, and *tet(*A/P*)* genes were not initially detected in either soil or manure. Detections between replicate soil or manure samples did not differ; *tet* genes were either detected in 100% of samples or not detected at all.

**TABLE 3:** Initial resistances in soil, manure slurry, and hydrated lime alkaline stabilized (HLAS) manure slurry assessed by multiplex PCR of tetracycline resistance genes and quantitative PCR of select resistance elements.

Mulitplex PCR, % positive†	Soil	Raw manure	HLAS-treated manure	Resistance mechanism/action
*tet(*A*)*	0	100	100	Efflux pump
*tet*(B*)*	0	100	100	Efflux pump
*tet*(C*)*	0	100	100	Efflux pump
*tet(*D*)*	100	0	0	Efflux pump
*tet(*E*)*	0	0	0	Efflux pump
*tet*(G)	100	100	100	Efflux pump
*tet(*K*)*	0	0	0	Efflux pump
*tet(*L*)*	100	0	0	Efflux pump
*tet(*A/P*)*	0	0	0	Efflux pump
*tet*(M*)*	0	100	100	Ribosome protection
*tet*(O*)*	0	100	100	Ribosome protection
*tet*(Q*)*	0	100	100	Ribosome protection
*tet(*S*)*	0	0	0	Ribosome protection
*tet*(X*)*	0	100	100	Antibiotic metabolism
Quantitative PCR‡				
**Per gram dry matte2r**				
16S rRNA gene	8.42 (0.08) A	8.43 (0.02) A	8.68 (0.04) B	Protein synthesis
*int*I1	3.48 (0.10) A	6.11 (0.02) B	6.15 (0.01) B	Integrase gene
*bla*_*CTX–M–*32_	3.80 (0.27) A	5.03 (0.03) B	4.76 (0.01) C	Antibiotic metabolism/ring cleavage
*sul(*I*)*	4.39 (0.07) A	6.70 (0.03) B	6.84 (0.03) C	Alternative dihydropteroate synthase
*erm(*B*)*	<1.7 A	6.65 (0.04) B	7.15 (0.02) C	Ribosome protection/dimethylation
**Log abundance normalized per 16S rRNA gene**				
*int*I1	−4.94 (0.05) A	−2.31 (0.04) B	−2.53 (0.05) B	
*bla*_*CTX–M–*32_	−4.78 (0.29) A	−3.40 (0.04) B	−3.92 (0.10) C	
*sul(*I*)*	−4.02 (0.05) A	−1.73 (0.05) B	−1.84 (0.07) B	
*erm(*B*)*	<−6.70 A	−1.78 (0.06) B	−1.53 (0.05) B	
**Abundance normalized per10^6^ 16S rRNA gene**				
*int*I1	12 A	4900 B	2980 B	
*bla*_*CTX–M–*32_	17 A	400 B	120 C	
*sul(*I*)*	96 A	18900 B	14700 B	
*erm(*B*)*	<0.2 A	16800 B	29800 B	

*†Results of multiplex PCR expressed as percentage of PCR-positive soil or manure samples (*n* = 3).*

*‡For quantitative PCR, each sample was amplified three times and averaged. The average log abundance (±SE) for three samples is reported. “ABC” denotes a difference (*P* < 0.05) between sample types (i.e., soil, manure, and HLAS-treated manure).*

From a quantitative perspective (Table 3), microbial abundance (based upon 16S rRNA genes per gram) in initial sources of raw manure and soil were similar (8.43 and 8.42 log abundance, respectively) with 16S rRNA genes in HLAS-treated manure slightly higher (8.68 log abundance) than the raw manure and soil. Comparing soil to manure, soil contained much lower (10- to 100-fold) abundances of *intI1, bla_*ctx–m–*32_*, and *sul(*I*)* compared to manure (raw and HLAS-treated). Furthermore, *erm(*B*)* was not detected (50 targets per gram detection limit) in soil but was detected in the manure at 6.65 and 7.15 log abundance in raw and HLAS-treated manure, respectively. Comparing manures, the abundance of *sul(*I*)* and *erm(*B*)* were slightly higher while *bla_*ctx–m–*32_* was slightly lower in HLAS-treated manure. Normalizing to the number of 16S rRNA genes, the differences between soil and manures remained consistent. Likewise, the significant differences observed between manures for *sul(*I*)* and *erm(*B*)*, when compared on a mass basis, were insignificant when normalized to 16S rRNA gene abundance.

### Temporal Trends in Soil/Manure Slurry Tetracycline Resistance

The multiplex PCR results revealed gene-specific persistence patterns (Table 4). Some genes were unaffected and consistently present over time. For instance, *tet(*M*)* and *tet(*O*)* genes originating in the manure were present with 100% frequency in all samples, regardless of treatment or time. Other *tet* genes [i.e., *tet(*E*), tet(*L*)*, and *tet(*S*)*] were detected very infrequently or only sporadically with no obvious differences attributed to soil moisture, manure treatment, or temporal trend. Other genes [i.e., *tet(*G*), tet(*K*), tet(*A/P*)*, and *tet(*Q*)*] detected initially or during the incubation were absent by Day 120 of the incubations. The frequency of *tet(*X*)* detection in soils receiving HLAS-treated manure was significantly lower than in samples receiving raw manure. Specifically, 75 to 100% of the soil reps receiving HLAS-treated manure were positive for *tet(*X*)* on Day 0, but just one of 24 reps (3.5%) were positive for *tet(*X*)* after Day 30. In comparison, the proportion of tests positive for *tet(*X*)* in soil receiving raw manure slurry after Day 30 (20 detections out of 24 tests, or 83%) was much greater (*P* < 0.0001). Similarly, the low-moisture soil (10% WHC) receiving raw manure retained *tet(*A*), tet(*B*)*, and *tet(*C*)* at a greater frequency (*P* < 0.001) during the incubation period (69, 87, and 100%, respectively, after Day 0) when compared to higher moisture soil receiving raw manure (6, 19, and 31%, respectively, after Day 0).

**TABLE 4:** Percent positive detection of *tet* genes in soil at 10 or 30% water holding capacity (WHC) with either raw manure or hydrated lime alkaline stabilized (HLAS) manure during simulated winter incubation using multiplex PCR*.

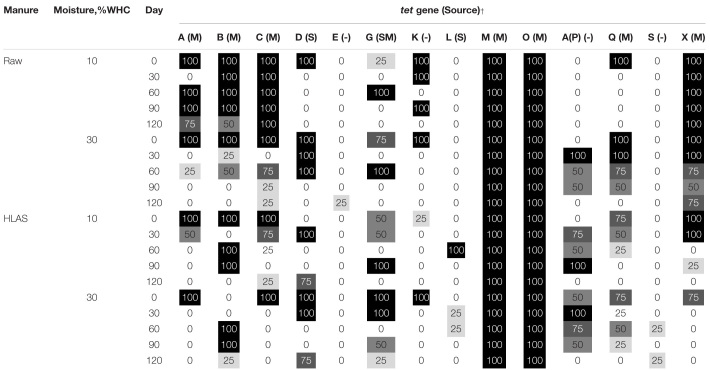

*Four replicates were analyzed for each manure/soil mixture. Statistical comparisons using the equality of proportions test (SAS) between cells can be calculated by the difference in percentage positive detections between two cells: 100% *P* = 0.005; 75% *P* = 0.028; 50% *P* = 0.102; 25% *P* = 0.285.

*†Source indicated by detection in soil (S), manure (M), both soil and manure (SM), or undetermined (−) as described in Table 3.*

*Shading indicates the percent positive detection representing 0% (white), 25% (light gray), 50% (medium gray), 75% (dark gray), and 100% (black) detection.*

Comparing *tet* resistance gene prevalence on the final day of the incubation, the occurrence of *tet(*M*)* and *tet(*O*)* were unaffected by manure treatment or soil moisture (100% of samples were positive). Manure treatment affected the frequency of detection of two *tet* resistance genes; *tet(*X*)* gene was less prevalent (0% in soil receiving HLAS-treated manure vs. 75% and 100% in the low and high moisture content soils, respectively, receiving raw manure; *P* ≤ 0.028), but *tet(*D*)* was more prevalent in soil receiving HLAS-treated manure (75% detection frequency) than in soil receiving raw manure (0% detection frequency); (*P* = 0.028) on Day 120. Soil moisture content alone did not yield a consistent effect on the frequency of *tet* resistance gene detection; however, the interaction of low soil moisture (10% WHC) and application of raw manure yielded greater frequency of *tet(*A*), tet(*B*)*, and *tet(*C*)* retention (detection frequencies of 75, 50, and 100%, respectively), in samples on Day 120. In comparison, *tet(A)*, *tet(*B*)*, and *tet(*C*)* were found at a frequency of 25% or less in other treatment combinations. Statistically significant differences between any two treatment combinations/sampling dates depended upon the difference in percentage positive. Differences between 25 or 50% detection frequencies were not significant (*P* = 0.285 and 0.102, respectively), while differences between 75 or 100% detection frequencies were significant (*P* = 0.028 and 0.005, respectively) based upon four replicates tested per treatment combination.

**FIGURE 2:**
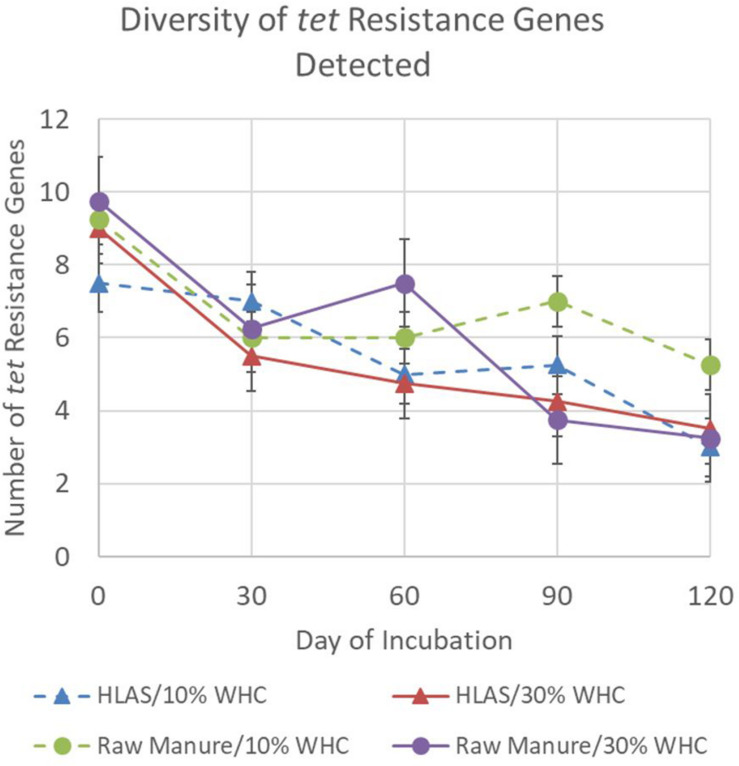
Number of different tetracycline resistance genes detected using the multiplex PCR method for the four treatment combinations, hydrated lime alkaline stabilized (HLAS) swine manure slurry (triangles) or raw swine manure slurry (circles) in soil originally at 10% (dashed line) or 30% (solid line) water holding capacity (WHC). Error bars = standard error.

Finally, the overall diversity of *tet* resistance genes among all treatment combinations declined during the simulated incubation period (Figure 2). The average number of different *tet* resistance genes detected in any treatment combination ranged from 7.5 to 9.75 on Day 0 but declined to a range of 3 to 5.25 by Day 120 (*P* < 0.001). Comparisons made on individual sampling days revealed that soil at initial 10% WHC receiving raw manure had greater diversity of *tet* resistance genes (*P* < 0.05) on Day 90 and 120 compared to all other treatments (*P* = 0.021). On Day 60, the diversity of *tet* resistance genes in soil receiving raw manure (at initial 10 and 30% WHC) were greater (*P* < 0.03) than in the soil receiving HLAS-treated manure.

### Temporal Trends in Resistance Elements and 16S rRNA Genes

**FIGURE 3:**
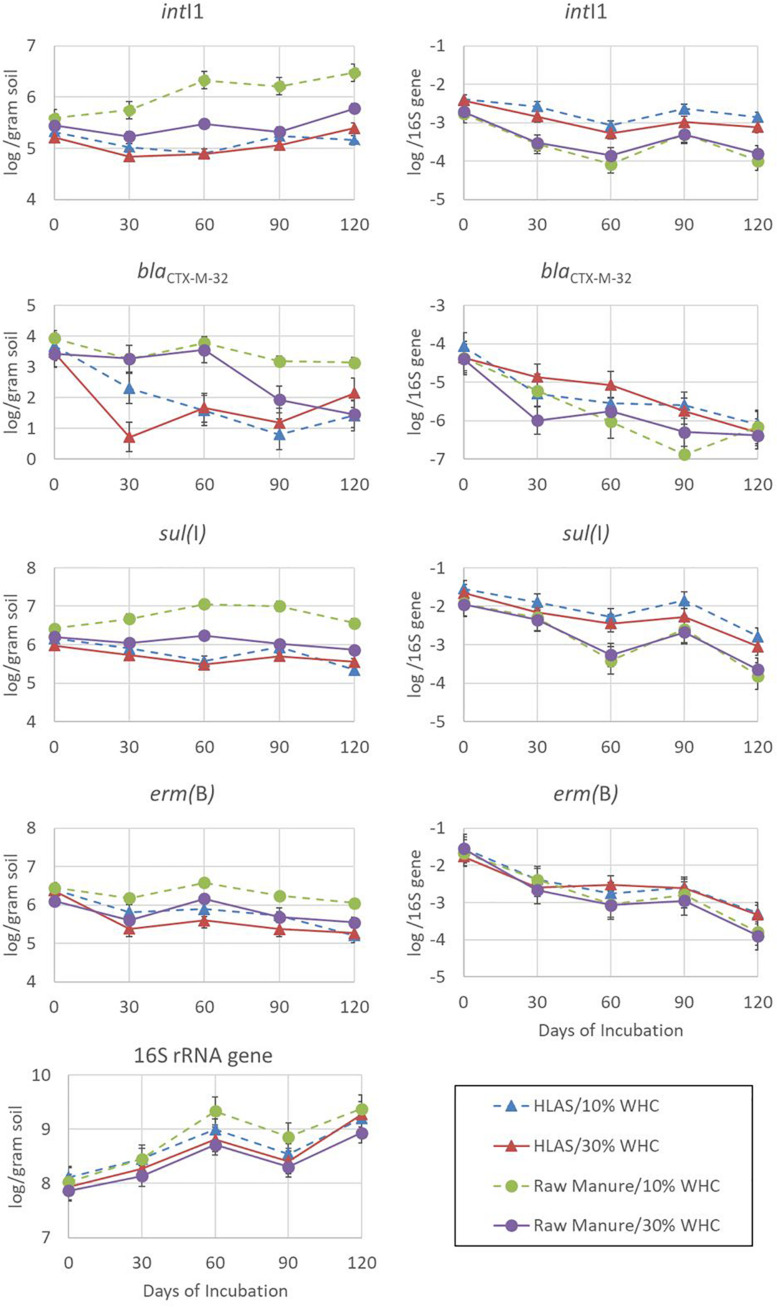
Abundance determined using quantitative polymerase chain reaction of resistance elements *int*I1, *bla*_*CTX–M–32*_, *sul(*I*)*, and *erm(*B*)* in addition to 16S rRNA genes over the 120-day simulated winter incubation. The four treatment combinations were hydrated lime alkaline stabilized (HLAS) swine manure slurry (triangles) or raw swine manure slurry (circles) in soil originally at 10% (dashed line) or 30% (solid line) water holding capacity (WHC) and are expressed in terms of log abundance per gram of dry soil (left-hand column of figures) or log abundance per 16S rRNA gene (right-hand column of figures). Error bars = standard error.

The abundances of *int*I1, *bla*_*CTX–M–*32_, *sul(*I*), erm(*B*)*, and 16S rRNA genes over the 120-day incubation period, when expressed on a per gram of dry slurry basis, varied among genes (Figure 3, left hand panels). Depending on the treatment combination, abundances could be (i) relatively stable [sul(I)], (ii) increasing over the incubation period (16S rRNA genes and intI1 in soil at 10% WHC receiving raw manure), (iii) decreasing slowly over the incubation period [erm(B)], or (iv) quite dynamic (blaCTX-M-32). Of the genes assessed, the abundance of the16S rRNA gene demonstrated the most consistent pattern (CV = 2.26 to 5.61%) regardless of treatments, increased through time, ranging from 7.86 to 8.11 log abundance copies per gram of dried manure/soil on Day 0 to 8.93 to 9.37 log abundance on Day 120.

Comparing among the four treatment combinations, soil at 10% WHC receiving raw manure yielded roughly 10-fold greater abundances of *int*I1, *bla*_*CTX–M–*32_, and *sul(*I*)* on Day 120 compared to all other treatment combinations. The result for *erm(*B*)* was consistent with other resistance genes and was 0.5 log abundance greater for soil at 10% WHC receiving raw manure than the other treatments. The abundance of resistance elements in soil receiving HLAS-treated manure was consistently lower throughout the incubation period compared to soils receiving raw manure; initial soil moisture did not have a large effect on abundance of resistance elements in soil receiving HLAS-treated manure. However, initial soil moisture did impact the abundance of resistance elements in soil receiving raw manure, with lower resistance gene abundances (*P* = 0.002) noted at the 30% WHC level compared to the 10% WHC level (5.54 versus 6.05 log abundance, respectively). There was no evidence of a similar synergistic effect for higher moisture soil receiving HLAS-treated manure.

Normalizing resistance elements to 16s rRNA genes helps to account for unequal initial microbial abundances and changes in the microbial population throughout the incubation. In this study, a very consistent pattern of decreasing resistance element abundance throughout the incubation period is evident (Figure 3, right hand panels). Although the abundance of an individual resistance element may have increased slightly during incubation (i.e., *int*I1 for soil at 10% WHC receiving raw manure), the proportion of the resistance element normalized to 16s rRNA genes in the microbial population decreased 10-fold (1 log). Interestingly, *int*I1 abundance in soil receiving HLAS-treated manure was only slightly reduced (roughly 0.5 log) relative to 16S rRNA. For resistance elements that showed decreasing abundance per gram of dried slurry [i.e., *erm(*B*)* and some *bla*_*CTX–M–*32_ treatments], the relative reduction was even greater (100-fold or 2 logs).

## Discussion

Manure injection is a common swine manure slurry application practice across the United States. When examined at a fine scale (<1–2 cm), this likely has a profound effect on the immediate soil environment and microbial processes. Considering the issue of antibiotic resistance, the narrow band of injected manure introduces a suite of very diverse *tet* resistance genes and resistance elements into the soil at abundances 10- to 100-fold greater than background soil concentrations. One might expect at very low winter-time temperatures that there would be limited microbial growth and very little microbial activity, having little impact on antibiotic resistance. However, studies on wheat straw decomposition at low temperatures indicate that although the rates of CO_2_ evolution are decreased, they are still substantial (Stott et al., 1986). In a more recent study, respiration rates measured at 0°C in agricultural and a humus forest soils from southern Sweden were positive and not predicted to reach zero respiration until −6°C (Pietikäinen et al., 2005). Supporting evidence from Arctic and Antarctic soils demonstrates that microorganisms mediate nutrient and carbon cycles at these very low temperatures (Eriksson et al., 2001; Kotsyurbenko, 2005). Stott et al. (1986) speculate that salts in soils may lower the freezing point of water enabling limited microbial decomposition. At the fine scale of manure injection bands in crop fields, this may be particularly important since swine manure slurries usually have high salinity and could depress the freezing point well below 0°C. The results of the simulated winter incubation presented herein, which included multiple freeze/thaw events, indicate that resistance in soil can be substantially altered even during the winter climate experienced in southwestern Minnesota, a region of extensive swine production.

Assessing a suite of tetracycline resistance genes through multiplex PCR reactions showed that both their occurrence and diversity were impacted by the simulated winter incubation. Ultimately, the presence of *tet* resistance genes on Day 120 may be the most important indicator of how soil moisture and manure treatment affect tetracycline resistance after a simulated winter incubation. From this perspective, the results are more easily interpreted. Particular *tet* resistance genes in the swine manure [*tet(*M*)* and *tet(*O*)*] showed no change after 120 days, yet others [*tet(*K*)*, *tet(*L*)*, *tet(*A/P*)*, and *tet(*Q*)*] present in the initial samples or occasionally detected during the incubation were no longer detected after 120 days. Furthermore, the persistence of other *tet* resistance genes [*tet(*A*)*, *tet(*B*)*, *tet(*C*)*, *tet(*D*)*, and *tet(*X*)*] were affected by application of HLAS-treated manure and/or initial soil moisture and were quite dynamic, disappearing entirely from some of the treatments. Finally, assessed *tet* resistance gene diversity illustrated a consistent decline in all the treatments during the simulated winter incubation, but less of a decline in the drier soil receiving raw manure. It is possible that the slightly lower moisture content provided conditions where manure microbes harboring *tet(*A*), tet(*B*)*, and *tet(*C*)* were able to persist within the soil microbial community. An alternative hypothesis is that the increased water content enhanced ice crystal formation during freezing, which increased the likelihood that manure bacteria were ruptured during multiple freeze/thaw events. Clearly additional research needs to be done to explore these hypotheses.

Converting the initial log abundances of resistance elements *int*I1, *bla*_*CTX–M–*32_, *sul(*I*)*, and *erm(*B*)* to numbers of targets observed per million copies of the 16S rRNA gene revealed that resistance elements in the soil were much less abundant (<100 copies) compared to those in manures, which contained hundreds to tens of thousands more resistance elements per million 16S rRNA genes (Table 3). Although, *int*I1, *bla*_*CTX–M–*32_, *sul(*I*)*, and *erm(*B*)* normalized to 16S rRNA abundance demonstrated a 10- to 100-fold reduction over the simulated winter incubation (Figure 3). A substantial portion of resistance element decrease could be attributed to the 10-fold increase in 16S rRNA abundance during the incubation. Although one would expect greatest increases in microbial abundance (i.e., 16S rRNA gene abundance) during the warmest periods (Day 0 to 30), the largest increases were observed from Day 30 to 60 and Day 90 to 120 when temperatures were slightly less than 0°C. One hypothesis that may explain these observations is that the series of freeze/thaw events (Day 0 to 30) lysed some microorganisms and/or released useful microbial substrates from manure solids. Cold tolerant species were then able to proliferate as substrates became available. A change in microbial community reflected in 16S rRNA sequence diversity would support this hypothesis but needs to be conducted.

The application of raw manure to soil at the lower WHC (10 vs. 30%) produced somewhat greater microbial abundance, which effectively diluted the relative abundance of most manure resistance elements for this treatment to a lower concentration. Somewhat to our surprise, even HLAS-treated manure also stimulated microbial growth. Although we presumed that the highly alkaline conditions in the manure would have reduced the capacity for microbial activity and survival, the soil environment was able to moderate the high pH in the treated swine slurry, and soil microbes were able to utilize the carbon and nutrient resources in the manure. For the resistance elements assessed using quantitative PCR in this study, nutrient addition did not preferentially stimulate proliferation of particular ARB over the simulated winter incubation, as was found by Udikovic-Kolic et al. (2014).

Specific *tet* resistance mechanisms in soil after the winter incubation were affected by swine manure application. Initially, only efflux pump types of resistance [*tet(*D*)*, *tet(*G*)*, and *tet(*L*)*] were detected in background soil, but application of swine manure introduced ribosome protection [*tet(*M*)*, *tet (*O*)*, and *tet(*Q*)*] and antibiotic metabolism [*tet(*X*)*] types of resistance to the soil environment. Although tet(Q) was no longer detectable by Day 120, the ribosomal protection resistance mechanism of *tet(*M*)* and *tet(*O*)* was still easily detected. Manure treatment also seemed to influence antibiotic metabolism resistance [*tet(*X*)*]. While *tet(*X*)* was still detected on Day 120 in soil receiving raw manure, *tet(*X*)* was no longer detectable in the soil receiving HLAS-treated manure. It is likely that the type of bacterial species harboring these *tet* resistances may be particularly sensitive to high pH and easily disrupted during HLAS.

The findings of these laboratory incubations present a unique look at changes in resistance at very low temperatures. However, these results are generally consistent with earlier studies investigating antibiotic resistance in swine manure slurry applied to soil and do not challenge consensus that the abundances of manure resistant genes may temporarily increase in the soil but decrease with time at seasonal scales. Even prior to swine manure application to soil, abundances of *tet* and *erm* genes in swine manure storage pits generally decrease by orders of magnitude as the fresh manure undergoes decomposition (Joy et al., 2014). A field study conducted by Garder et al. (2014) in Iowa examined swine manure injected into soil and may offer the closest comparison to the research presented here. The Iowa study collected data seasonally and found that the absolute abundance (per gram soil) of *erm(*B*)* and *erm*(F) decreased by 5 – 6 log abundance over a year. At the shortest time step, fall to spring, *erm(*F*)* absolute abundance decreased by 2 – 3 log per gram soil. Although over a short time period (65 days) and at higher temperature (25°C), another study conducted in a greenhouse found *tet(*C*)* and *tet(*Z*)* decreased from <0.5 to 2 log abundance after swine manure was initially added to soil (Kang et al., 2017). Finally, a recent study conducted using dairy manure investigated how resistance elements respond to freeze/thaw and various WHC (25 to 75%) at low (5°C) temperature over a 56-day incubation (McKinney and Dungan, 2020). Examining *int*I1, *sul(*I*)*, *tet(*M*)*, *erm(*B*)*, and 16S rRNA genes, they were unable to detect erm(B) but found decreasing absolute abundance (per gram soil) of all other resistance elements at lowest temperature and for all resistance elements except for *tet(*M*)* which did not change at the lowest WHC. Freeze/thaw had little effect on resistance element absolute abundance except for *sul(*I*)* which declined slightly. One subtle difference with our findings was that 16S rRNA gene abundance did not change during the incubation at lowest temperatures (5°C) and WHC (25%). It is interesting to note that 16S rRNA abundance was already above 9 log abundance per gram soil while the abundance in this study started at 8 log abundance and ended near 9 log abundance. Would the 16S rRNA abundance in our study have continued to increase with a longer incubation time at 0°C or would it have plateaued?

Initially, we hypothesized that HLAS treatment would have a strong effect on resistance by directly affecting the viability of microorganisms, but that incubation time would yield minimal changes in resistance after the imposition of “winter” incubation conditions due to low microbial and enzymatic activity. However, our results demonstrate that the soil community even at low temperatures is quite responsive to manure nutrient input. Furthermore, resistance elements were surprisingly dynamic during a presumed inactive period.

## Conclusion

Swine manure application introduced a diverse set of *tet* resistance genes into low *tet* resistance diversity agricultural soil, increasing the diversity from three of fourteen assessed genes to ten different *tet* genes in the mixed manure/soil. During a simulated winter incubation, the diversity of tetracycline resistance genes declined. However, two swine manure associated resistance genes [*tet(*M*)* and *tet(*O*)*] persisted. The persistence of other *tet* resistance genes [*tet(*A*)*, *tet(*B*)*, *tet(*C*)*, *tet(*D*)*, and *tet(*X*)*] were affected by HLAS treatment and initial soil moisture. Other resistance elements [*int*I1, *bla*_*CTX–M–*32_, *sul(*I*)*, and *erm(*B*)*] normalized to 16S rRNA abundance demonstrated a 10- to 100-fold reduction over the simulated winter incubation, due in part to the 10-fold increase in 16S rRNA abundance, and to a limited reduction of most resistance element abundances.

## Data Availability Statement

The raw data supporting the conclusions of this article will be made available by the authors, without undue reservation.

## Author Contributions

DM was the principal scientist responsible for conducting the analysis of soil and manure samples, reviewing statistical analyses, developing figures and tables, and finishing the final drafts of the manuscript. MJ was an undergraduate student and responsible for conducting lab analyses and preparing initial statistics, figures, and drafts of the manuscript. LD contributed to laboratory analysis and manuscript preparation. AS was responsible for conducting the incubations and revising manuscripts. All authors contributed to the article and approved the submitted version.

## Conflict of Interest

The authors declare that the research was conducted in the absence of any commercial or financial relationships that could be construed as a potential conflict of interest.
